# SARS-CoV-2 in children and their accompanying caregivers: Implications for testing strategies in resource limited hospitals

**DOI:** 10.1016/j.afjem.2022.04.007

**Published:** 2022-04-25

**Authors:** Liezl Smit, Andrew Redfern, Sadia Murray, Juanita Lishman, Marieke M. van der Zalm, Gert van Zyl, Lilly M. Verhagen, Corné de Vos, Helena Rabie, Annemarie Dyk, Mathilda Claassen, Jantjie Taljaard, Marina Aucamp, Angela Dramowski

**Affiliations:** a Department of Paediatrics and Child Health, Stellenbosch University, Cape Town, South Africa; b Desmond Tutu TB Centre, Department of Paediatrics and Child Health, Stellenbosch University, Cape Town, South Africa; c Division of Medical Virology, Stellenbosch University, Faculty of Medicine and Health Sciences and National Health Laboratory Services, Tygerberg business unit, Cape Town, South Africa; d Section of Pediatric Infectious Diseases, Laboratory of Medical Immunology, Radboud University Medical Center, Radboud Center for Infectious Diseases, Nijmegen, The Netherlands; e Department of Paediatric Infectious Diseases and Immunology, Amalia Children’s Hospital, Radboud University Medical Center, Nijmegen, The Netherlands; f Department of Paediatric Surgery, Stellenbosch University, Cape Town, South Africa; g Division of Medical Virology, Stellenbosch University, Faculty of Medicine and Health Sciences and National Health Laboratory Services, Tygerberg business unit, Cape Town, South Africa; h Division of Infectious Diseases, Department of Medicine, Tygerberg Hospital and Stellenbosch Univerisity, Cape Town, South Africa; i Unit for Infection Prevention and Control (UIPC), Tygerberg Hospital, Cape Town, South Africa

**Keywords:** SARS-CoV-2, Symptom screening, Children and accompanying caregivers, SARS-CoV-2, Severe Acute Respiratory Syndrome Coronavirus 2, COVID-19, Coronavirus-19

## Abstract

**Background:**

Identification of SARS-CoV-2 infected individuals is imperative to prevent hospital transmission, but symptom-based screening may fail to identify asymptomatic/mildly symptomatic infectious children and their caregivers.

**Methods:**

A COVID-19 period prevalence study was conducted between 13 and 26 August 2020 at Tygerberg Hospital, testing all children and their accompanying asymptomatic caregivers after initial symptom screening. One nasopharyngeal swab was submitted for SARS-CoV-2 using real-time reverse-transcription polymerase chain reaction (rRT-PCR). An additional Respiratory Viral 16-multiplex rRT-PCR test was simultaneously done in children presenting with symptoms compatible with possible SARS-CoV-2 infection.

**Results:**

SARS-Co-V 2 RT-PCR tests from 196 children and 116 caregivers were included in the analysis. The SARS-CoV-2 period prevalence in children was 5.6% (11/196) versus 15.5% (18/116) in asymptomatic caregivers (*p*<0.01). Presenting symptoms did not correlate with SARS-CoV-2 test positivity; children without typical symptoms of SARS-CoV-2 were more likely to be positive than those with typical symptoms (10.2% [10/99] vs 1% [1/97]; *p*<0.01). Children with typical symptoms (97/196; 49.5%) mainly presented with acute respiratory (68/97; 70.1%), fever (17/97; 17.5%), or gastro-intestinal complaints (12/97; 12.4%); Human Rhinovirus (23/81; 28.4%) and Respiratory Syncytial Virus (18/81; 22.2%) were frequently identified in this group. Children-caregiver pairs’ SARS-CoV-2 tests were discordant in 83.3%; 15/18 infected caregivers’ children tested negative. Symptom-based COVID-19 screening alone would have missed 90% of the positive children and 100% of asymptomatic but positive caregivers.

**Conclusion:**

Given the poor correlation between SARS-CoV-2 symptoms and RT-PCR test positivity, universal testing of children and their accompanying caregivers should be considered for emergency and inpatient paediatric admissions during high COVID-19 community transmission periods. Universal PPE and optimising ventilation is likely the most effective way to control transmission of respiratory viral infections, including SARS-CoV-2, where universal testing is not feasible. In these settings, repeated point prevalence studies may be useful to inform local testing and cohorting strategies.

## African Relevance

• Children and their accompanying caregivers pose a unique challenge in emergency centres and admission wards, as either may pose a risk of transmission of SARS-CoV-2, especially in low and middle income countries (LMICs) with limited laboratory testing capacity, few isolation rooms, open wards and limited personal protective equipment (PPE) resources.
• Hospitals in LMICs may further have limited capacity to alter normal patient flow patterns and cohorting strategies to accommodate potentially infectious patients during the Covid-19 pandemic.
• Symptom-based screening is done to identify possible SARS-CoV-2 infection, but this screening method failed to identify 90% of children and 100% of caregivers with positive SARS-COV-2 PCR results in our study.
• Discordant test results in 83% of child-caregiver pairs further made testing of either the child or the caregiver unreliable in excluding infection of the other.
• Where universal PCR testing of children and their accompanying caregivers is not feasible, repeated point prevalence studies may be useful to inform local testing and cohorting strategies.

## Introduction

Identification of Severe Acute Respiratory Syndrome Coronavirus 2 (SARS-CoV-2) infected individuals at the time of hospital presentation is imperative to inform isolation and transmission-based precautions and avoid healthcare-associated transmission [1]. Children and their accompanying caregivers pose a unique challenge, as either may pose a risk of transmission, especially in low and middle income countries (LMICs) with few isolation rooms, open wards and limited personal protective equipment (PPE) resources [2,3]. Many low resource settings also have over-crowded emergency centres and scarce critical care and even basic care resources, including heathcare workers [4], making cost-effective and appropriate use of SARS-CoV-2 testing a critical part of the medical and infection, prevention and control preparedness of these healthcare facilities [5] .

Symptom screening has been adopted to identify individuals with possible SARS-CoV-2 infection for targeted laboratory testing in South African workplaces as part of the Coronavirus-19 (COVID-19) disease pandemic response [6,7]. Asymptomatic / early infection phase transmission however plays an important role in the spread of SARS-CoV-2, complicating infection control strategies in congregate settings like emergency centres or open wards [8], [9], [10]. Children with SARS-CoV-2 may further present with a number of non-specific symptoms rather than the more ‘typical’ symptoms such as cough, fever, sore throat, myalgia, and diarrhoea [11,12]. These symptoms overlap considerably with non-SARS-CoV-2 related illnesses and other respiratory viruses, which form a large proportion of emergency departments’ paediatric admissions [13]. The recent surge in non-SARS-CoV-2 respiratory infections seen in many countries makes it even more important to have appropriate logistics in place to discriminate between SARS-CoV-2 and non-SARS-CoV-2 related illnesses. [14,15]. This lack of specificity of signs or symptoms, and the substantial proportion of asymptomatic or mild infections in children, hampers the reliability of symptom-based screening for identification of SARS-CoV-2 in children attending hospital.

In order to identify those with SARS-CoV-2 infection, universal laboratory testing of all children and their accompanying caregivers may be required, but this strategy could overload healthcare resources, particularly in LMICs such as South Africa. Given the concerns regarding the use of symptom-based SARS-CoV-2 testing strategies in children and their caregivers, we conducted a 2-week period prevalence study in all children and their asymptomatic caregivers presenting to the paediatric department of a large teaching hospital in Cape Town, South Africa, to inform testing and patient cohort strategies towards the end of a COVID-19 epidemiological wave.

## Methods

### Study setting and design

Tygerberg Hospital is a 1384 bed teaching hospital affiliated to Stellenbosch University, with 200 beds dedicated to paediatric medical and surgical care which look after children up until the age of 13 years. From 13 to 26 August 2020, a period prevalence surveillance study was conducted where all children and their accompanying caregivers were tested for SARS-CoV-2 at the Departments of Paediatrics and Child Health and Paediatric Surgery at Tygerberg Hospital, Cape Town, South Africa. This included all emergency presentations to the Paediatric Emergency Department and elective admissions to both the paediatric medical and surgical wards. Previous COVID-19 infection status was not routinely assessed as part of the admission. Both children and caregivers were screened based on the suspected COVID-19 case definition [16,17] for symptom(s) typically reported of SARS-CoV-2 infection (fever, acute onset cough, runny nose or sore throat, loss of smell or taste, flu-like symptoms, gastro-intestinal symptoms e.g. diarrhoea and vomiting or abdominal pain) at presentation and daily during admission on symptom screening forms. Symptomatic caregivers of children requiring admission were asked to isolate at home. All asymptomatic caregivers were offered a voluntary test for SARS-CoV-2 at the time of admission of their child. They were not refused entry to the wards if they declined to be tested. Caregivers were initially tested with their child on admission, but due to the high test-burden caregivers were referred to the COVID-19 testing site on the hospital premises in week two of the study. This lead to some caregivers declining the test. Children and caregivers already admitted prior to the surveillance period or neonates and their caregivers admitted to the neonatal wards were excluded.

South Africa was placed under national level 5 lockdown on 23 March 2020. A gradual and phased easing of the lockdown restrictions began in May 2020, with an amended alert level 2 in place during this study [18]. During this period 149 paediatric cases per week were recorded in the Western Cape Province from a high of 505 in July 2020 [19].

Study approval was granted by the Human Health Research Ethics Committee (HREC N20/04/013) of the Faculty of Medicine and Health Sciences, Stellenbosch University.

*SARS-CoV-2 laboratory infection control strategies* at Tygerberg Hospital during the peak of the COVID-19 epidemiological wave included daily symptom screening of children, their accompanying caregivers and healthcare workers, a restricted hospital visitor policy, mandatory cloth masks for all caregivers at all times, and universal masking of staff with use of visors, aprons and gloves when providing direct patient care. N95 respirators were used by health care workers (HCWs) where children or caregivers tested positive, or where aerolizing procedures were performed in children whose SARS-CoV-2 status was not yet known but who were considered as high risk for possible SARS-CoV-2 infection. Owing to limited availability of isolation rooms, plastic room dividers were installed between beds in all open wards. Children with symptoms suggestive of SARS-CoV-2 were admitted to dedicated COVID-19 Person under investigation (PUI) areas, which included the Emergency Unit short stay ward, for isolation or cohorting until test results became available. At the time of the study the National Health Laboratory Service (NHLS) Virology Laboratory at Tygerberg Hospital offered ‘ultra-urgent’ SARS-COV-2 PCR testing for all hospitalised PUI patients with turnaround time (TAT) of 2–3 h during daytime and 5–6 h at night. TAT for non-urgent tests were 24 h. Bedside rapid tests were not available in the public sector at the time. Children with negative tests and those without symptoms suggestive of SARS-CoV-2 infection, were admitted from the dedicated COVID-19 PUI areas to dedicated non-COVID-19 wards. A child-caregiver pair was admitted to the COVID-19 ward if either of them tested positive.

Laboratory analysis: One nasopharyngeal swab (NPS) for SARS-CoV-2 real-time reverse-transcription polymerase chain reaction (rRT-PCR) was collected from all newly admitted paediatric patients and asymptomatic caregivers who agreed to be tested. A laboratory-confirmed case of COVID-19 was defined as a positive SARS-CoV-2 RT-PCR test. Due to supply challenges at the height of the pandemic three different assays were used: the Allplex 2019nCOV / Allplex SARS-CoV-2 assay (Seegene, South Korea) RT PCR with 3 targets: N, E and RdRp positive if 2 or more targets are present and inconclusive if a single target has a Ct > 37; negative if no signal in all targets), the TaqPath COVID-19 CE-IVD RT-PCR kit (Thermo Fisher) with 3 targets: ORF-1, Spike protein and N; positive target Ct <= 37, as well as the Xpert Xpress SARS-CoV-2 (Cepheid) with dual target N and E; for single positive target threshold is Ct 38. All assays were verified according to NHLS policies with South African National Accreditation System (SANAS) accreditation ISO 17025. These assays had similar diagnostic sensitivity despite some variability in analytical sensitivity [20].

An additional nasopharyngeal swab (NPS) for Respiratory Viral 16 (RV-16) multiplex rRT-PCR testing (Anyplex™ II RV16 Detection assay, Seegene, South Korea) [21], was done in those children presenting with symptom(s) typically reported of SARS-CoV-2 infection. A respiratory panel test was also requested in asymptomatic children who tested positive for SARS-CoV-2.

### Data sources

Demographic and clinical data, including age, gender, presenting symptoms or reason for hospital admission, human immunodeficiency virus (HIV) status and NPS results were collected prospectively and completed with information from patient discharge summaries (retrospectively) if incomplete.

### Statistical analysis

Children were categorised into two groups: those presenting *with* symptoms of SARS-CoV-2 infection typically reported in the literature [16,17] and those *without*. Children presenting *with* typical symptoms were sub-categorised according to their main presenting symptoms; ‘acute respiratory’, ‘fever’, or ‘gastro-intestinal’. These summary categories are in alignment with presenting symptoms in children with possible SARS-Cov-2 infection and were chosen for analysis rather than the listing of individual complaints. Children *without* typical symptoms were sub-categorised as ‘surveillance’ or ‘pre-procedure’ if admitted for elective surgery/interventions. Child and caregiver pairs were grouped together to compare their SARS-CoV-2 results. Data was processed and analysed utilising descriptive statistics, calculating the frequency distribution of variables and measuring central tendency and spread. Differences between groups were calculated using t-tests and chi-square tests as appropriate. Statistical significance was set at a *p*-value of <0.05. Stata version 15 (Stata corp version 27., College Station, Texas, USA) was used for data analysis.

## Results

In total three hundred and twenty four SARS-CoV-2 tests were performed during the two-week period prevalence study with an average of 23 tests per day. Three child-caregiver pairs and six tests in children were excluded from the analysis due to testing being performed outside of the study period, or an inability to verify laboratory results. Four children included in the analysis had two episodes of SARS-CoV-2 RT-PCR testing during the study period, three from oncology and one child who was re-admitted for bronchoscopy. The number of tests included in the final analysis were 196 in children and 116 in caregivers.

The median age of all children was 19.3 months (interquartile range “IQR” 4.6–65.4) with 54.6% (107/196) males; 1.5% (3/196) children were HIV-infected, 60.4% (119/196) HIV-negative, with HIV status unknown in 38.1% (75/196) children (Table 1). The SARS-CoV-2 period prevalence in admitted children was 5.6% (11/196) compared to 15.5% (18/116) in accompanying asymptomatic caregivers (*p*<0.01). Almost half (49.5%; 97/196) of the children presented *with* symptoms typical of SARS-CoV-2 infection. These children were younger with a median age of 13.3 months (IQR 3.1–39.7), compared to 33.9 months (IQR 9.2–86.7) in children presenting *without* typical symptoms (*p*<0.01). Presence of suggestive COVID-19 symptoms did not correlate with SARS-CoV-2 test positivity; in fact, children *without* typical symptoms were more likely to be SARS-CoV-2 positive than those *with* typical symptoms (10.2% [10/99] vs 1.0% [1/97]; p<0.01).

**Table 1:** Demographic and clinical data of ALL children included in the SARS-CoV-2 period prevalence study at Tygerberg Hospital 13 – 26 August 2020.

Category	All tests	*With*^a^ symptoms typical of SARS-CoV-2 infection	*Without*^b^ symptoms typical of SARS-CoV-2 infection	*p*-value
Number (%)	196 (100%)	97 (49.5%)	99 (50.5%)	-
Age in months; median (IQR)	19.3 (4.6–65.4)	13.3 (3.1–39.7)	33.9 (9.2–86.7)	<0.01
Gender; male n (%)	107 (54.6%)	50 (51.5%)	57 (57.6%)	0.72
HIV status;				
n (%)	3 (1.5%)	2 (2.29%)	1 (1.0%)	0.57^c^
Positive	119 (60.4%)	49 (49.5%)	70 (71.4%)	
Negative	74 (37.8%)	47 (48.5%)	27 (27.3%)	
Unknown				
SARS-CoV-2;				
n (%)	11 (5.6%)	1 (1.0%)	10 (10.2%)	<0.01
RT-PCR positive				

a fever, acute onset cough, runny nose or sore throat, loss of smell or taste, flu-like symptoms, gastro-intestinal symptoms e.g. diarrhoea and vomiting or abdominal pain

b other acute or elective reason for admission

c children with unknown status were excluded from this analysis

In children presenting *with* typical symptoms of SARS CoV-2 infection, acute respiratory symptoms (70.1%; 68/97) were the most common typical presenting complaint followed by fever (17.5%; 17/97) and gastro-intestinal (12.4%; 12/97) (Table 2). The one child who tested SARS-CoV-2 positive in this group – a 10 year old boy with acute myeloid leukaemia – presented with a combination of fever, flu-like symptoms, runny nose and diarrhoea. In 83.5% (81/97) of children presenting *with* typical symptoms a RV16 viral panel result was available. Human Rhinovirus (HRV) (28.4%; 23/81) and Respiratory Syncytial Virus (RSV) (22.2%; 18/81) were the dominant single viral pathogens detected. In 25.0% (20/81) of children more than one virus was detected with RSV and HRV the dominant co-pathogens (11%; 9/81). No co-pathogens were detected on the RV16 viral panel in the one SARS-CoV-2 positive child.

**Table 2:** Demographics and viral testing results of children *with* symptoms typical of SARS-CoV-2 infection, categorised by main presenting symptom

Main Presenting Symptom^a^	ALL	Acute Respiratory	Fever	Gastro-intestinal	*p*-value
Number (%)	97 (100%)	68 (70.1%)	17 (17.5%)	12 (12.4%)	-
Age in months; median (IQR)	13.3 (3.1–39.7)	6.3 (2.7–34.3)	28.5 (20.9–64.5)	14.3 (8.1–29.6)	0.373
Gender; Male n (%)	50/97 (51.5%)	37/68 (54.4%)	7/17 (41.2%)	6/12 (50.0%)	0.617
SARS-CoV-2 RT-PCR positive, n (%)	1/97 (1.0%)	1/68 (1.4%)	0/17 (0%)	0/12 (0%)	-
RV16 PCR viral panel results; n (%)					
not done/missing	16/97 (16.4%)	11/68 (16.2%)	3/17 (17.6%)	2/12 (16.7%)	
negative	20/81 (25.0%)	10/57 (17.5%)	6/14 (42.9%)	4/10 (40.0%)	-
single virus detected	43/81^b^ (53.1%)	33/57 (57.9%)	6/14 (42.9%)	4/10 (40.0%)	
>1 virus detected	18/81^c^ (22.2%)	14/57 (24.5%)	2/14 (14.3%)	2/10 (20.0%)	

a Patients could present with one or more symptoms, but for this analysis, patients were categorized into 3 groups based on the main presenting complaint.

b Single viruses detected (total 43/81[53.1%]), broken down as follows: 23/43(53.5%) human Rhinovirus A/B/C (HRV); 18/43(41.9%) Respiratory syncytial virus A/B (RSV); 1/43(2.3%) Adenovirus (AdV); 1/43(2.3%) human Bocavirus 1-4 (HBoV 1-4)

c >1 virus detected (total 18/81[11.1%]), broken down as follows: 9/18(50.0%) HRV + RSV; 2/18 (11.1%) HRV + AdV; 2/18 (11.1%) HRV + HBoV; 1/18 (5.6%) RSV + HBoV; 1/18 (5.6%) HRV, AdV + 229E (human Coronavirus); 1/18 (5.6%) HRV, AdV + HBoV; 1/18 (5.6%) HRV, RSV + HEV (human Enterovirus); 1/18(5.6%) HRV, RSV + A

In the 50.5% (99/196) of children presenting *without* typical symptoms of SARS-CoV-2 infection, 38.4% (38/99) were electively admitted for procedures with potential risk of aerosol-generation e.g. bronchoscopy or intubation; 10.5% (4/38) of these asymptomatic children tested positive for SARS-CoV-2. In total, 10.2% (10/99) children who had no symptoms typical of SARS-CoV-2 infection and who were admitted directly to the non-COVID-19 areas, had a positive SARS-CoV-2 test: 50.0% (5/10) were admitted to the surgical ward, 20.0% (2/10) to oncology, and one each to the rheumatology, gastro-intestinal and general paediatrics wards. Only 20.0% (2/10) caregivers tested positive for SARS-CoV-2 in this group; 50.0% (5/10) tested negative, and 30.0% (3/10) were not tested. A RV16 viral panel result was available for all 10 of the SARS-CoV-2 infected patients in this group, with co-pathogens detected in 50.0% (5/10); HRV being the most common virus detected (80.0%, 4/5).

One hundred and sixteen of the possible 196 (59.2%) asympomatic caregivers volunteered for SARS-CoV-2 testing and 15.5% (18/116) tested positive. Children and caregiver SARS-CoV-2 tests were discordant in 83.3%, with 15/18 infected caregivers’ children testing negative. Only 27.3% (3/11) of the SARS-CoV-2 positive children's caregivers tested positive for SARS-CoV-2. One positive mother had 2 children admitted with only 1 testing positive for SARS-Cov-2; her other child remained asymptomatic and tested negative for SARS-CoV-2.

## Discussion

This SARS-CoV-2 surveillance study from an African country confirms the limitations of symptom screening in identifying both children and their accompanying caregivers with SARS-CoV-2, and the value of SARS-CoV-2 testing of all children and their accompanying caregivers regardless of COVID-19 case definition/ suspicion to inform hospital-based infection control strategies. The low paediatric SARS-CoV-2 positivity rate of 5.6% during the study period reflected the overall decline in COVID-19 incidence in the Western Cape Province following the pandemic peak in June/July 2020, which was substantially lower than that documented in earlier months of the pandemic or during subsequent waves[19].

Despite symptom screening being recommended, distinguishing between truly asymptomatic or pre-symptomatic patients remains challenging [22], [23], [24]. Similar to our report, a Republic of Korean study [22] reported that symptom screening failed to identify most of their paediatric SARS-CoV-2 cases, with 22% of children being asymptomatic at presentation and remaining asymptomatic after COVID-19 diagnosis. These children were tested when in close contact with confirmed cases, epidemiologically linked to COVID-19 outbreaks, arrived from abroad, or had symptoms suspicious of COVID-19 as judged by doctors. In a Canadian cohort of 2 463 children [23] tested because of high risk exposure or epidemiologically linked to COVID-19 outbreaks, a 35.9% SARS-CoV-2 positivity rate was reported in asymptomatic children. In the Republic of Korea's cohort of 91 patients, with a median age of 10 years, respiratory (60%), followed by gastro-intestinal symptoms (18%) were the most common presenting complaints [22], in keeping with our findings. Loss of smell and taste were further common (16%) in this cohort, whereas these symptoms were not reported in our study population, likely owing to the younger age of our cohort in whom these symptoms may go unrecognized. Respiratory symptoms were similarly the most commonly reported symptoms in the Canadian cohort [23], which was not found to be predictive of positive SARS-CoV-2 results.

Asymptomatic elective admissions had the highest SARS-CoV-2 positivity rate in our study.

This is in contrast with point prevalence studies from Japan [24], Canada [25] and Italy [26] that reported low positivity rates varying from 0.03% to 0.4% in asymptomatic individuals admitted for elective procedures or presenting to emergency departments. These results most likely reflected the low level of community transmission in the settings during these studies. The cost-effectiveness and labour intensity of universal PCR testing could thus be questioned during periods of low community transmission.

Respiratory viruses other than SARS-CoV-2 were detected more frequently in children with symptoms typical of SARS-CoV-2 infection in our study, with HRV and RSV being the most prevalent viruses. Among SARS-CoV-2 infected children, co-infection with other viruses was detected in more than half, comparable to findings from China where 19/34 (51.4%) patients showed co-infection with pathogens other than SARS-CoV-2 [27]. The high rate of RSV detection coincided with the surge in RSV infections identified in South Africa after the COVID-19 pandemic peak when lockdown measures were lifted [28]. This is in keeping with studies reporting a reduction of non-SARS-CoV-2 seasonal illnesses during periods of strict social restrictions as part of non-pharmaceutical interventions against COVID-19 [29,30] followed by an increased prevalence once social restrictions are eased [31], [32], [33], [34], [35]. This further highlights how difficult it is to distinguish children with SARS-CoV-2 infection from infection with other common respiratory viruses with the additional changes in patterns of non-SARS-CoV-2 infection transmission brought on by lockdown measures. On the other hand the detection of both SARS-CoV-2 and other viruses in asymptomatic children in our study underlines the real risk of underdetection of asymptomatic / early infections with possible transmission.

The importance of adult caregivers as possible transmitters of SARS-CoV-2 within paediatric services has been highlighted before [36]. There is a paucity of published reports of caregiver SARS-CoV-2 surveillance testing in paediatric units in Africa, but up to 80% of adults with SARS-CoV-2 are reportedly asymptomatic [37] which might be higher than in children [3,38]. Our findings support this, as the SARS-CoV-2 positivity rate was higher in asymptomatic adult caregivers than their children. Similarly Hassoun and colleagues [3] reported a higher positivity rate in *asymptomatic* caregivers (7.5%) compared to *asymptomatic* children (2.5%) during a point prevalence study at a pediatric emergency department in New York. Their reported caregiver-child SARS-CoV-2 RT PCR test concordance of 95% was however much higher than our study findings. Although children less than 10 years of age may be less likely to cause nosocomial infection spread [1,39], risk of transmission remains of concern in LMICs where overcrowded emergency centres, the lack of isolation rooms and use of large cohort rooms for admission of multiple child-caregiver pairs may contribute to healthcare-associated COVID-19 transmission [40,41]. Caregiver transmission risk needs specific attention when designing paediatric COVID-19 testing strategies and infection control measures in hospitals.

A lack of both human and laboratory resources in LMICs may limit the implementation of universal SARS-CoV-2 testing of hospitalised children and their caregivers. The risk of false negative PCR tests during the virus incubation period makes a pre-admission testing strategy alone not sufficient to prevent nosocomial transmission infection, with guidelines recommending re-screening of admitted patients after 5–7 days [42,43]. Low cost, point of care antigen tests have potential advantages, especially in emergency settings, and should be considered. [25,44]. However, antigen testing may be able to distinguish children with a high viral load who may be infectious, but are overall less sensitive in children compared to adults, making a negative test potentially unreliable [45]. Appropriate social distancing, hand hygiene, environment surface cleaning, isolation of infected people and universal face-masking remain the best strategies to avoid spread of infection [46], as patient cohorting according to symptoms seems unreliable. Health systems in LMICs are under-resourced and in many settings may be unable to effectively implement these public health measures [47]. Repeated local period prevalence surveys could be used to inform local testing strategies and assist with the implementation of effective IPC activities and appropriate resource allocation [48].

Universal PCR testing of both children and caregivers presenting to the emergency unit were not feasible at Tygerberg Hospital, with only children requiring admission to wards tested for SARS-CoV-2 irrespective of presenting symptoms or complaints. Universal IPC precautions were rather implemented, with only SARS-CoV-2 positive children cohorted in a dedicated ward. Local prevalence data will continue to inform future testing and cohort strategies.

Limitations of this study are the single centre, small sample size and timing of the surveillance period at the “tail end” of a pandemic wave. Another limitation may be the possibility of false negative test results. Several factors may contribute to a lack of RT-PCR-testing sensitivity including the amount of material sampled and the assay sensitivity [49]. As some data were collected retrospectively, variables may be missing or not documented. Strengths of this study are the inclusion of testing for other respiratory viruses in symptomatic children and the inclusion of testing results for asymptomatic caregivers.

## Conclusion

Symptom-based COVID-19 screening is inadequate to identify SARS-CoV-2 infections in children and caregivers. Changes in epidemiological patterns of non-SARS-CoV-2 infection transmission influenced by changes in lockdown measures as part of the COVID-19 pandemic response further complicates this symptom-based approach. Both asymptomatic PCR-positive children and caregivers pose a potential risk of SARS-CoV-2 transmission to staff and patients in open wards. This has important implications for patient flow and cohorting strategies. To mitigate COVID-19 infection transmission risk, all hospitalised children and their caregivers should ideally be tested for SARS-CoV-2 on admission during the pandemic, irrespective of symptoms. In resource-limited settings where testing of all admitted children and their caregivers may not be possible, universal IPC precautions should be implemented, as cohorting according to symptoms is unreliable. Regular prevalence studies of admitted child-caregiver pairs may be helpful to inform revision of local COVID-19 testing strategies in combination with local community prevalence data.

## Dissemination of Results

Results from this point prevalence study was shared with the Department of Paediatrics and Child Health staff during a COVID-19 academic session. The results were also shared with the Tygerberg Hospital management team, and specifically the COVID-19 hospital response team.

## Authors’ Contribution

Authors contributed as follows to the conception of the work; the acquisition, analysis, or interpretation of data for the work; and drafting the work or revisiting it critically for important intellectual content: LS contributed 15%; SM, AR, LMV, JL, HR, MvdZ and AD 10%, CdV 5% and GvZ, MC, JT, AD and MA 2% each. All authors approved the version to be published and agreed to be accountable for all aspects of the work.

## Funding

This work was supported by an unrestricted SEED funding from Stellenbosch University special vice-rector fund for COVID-19 research. Angela Dramowski is supported by a National Institutes of Health Emerging Global Leader Award (NIH K43 TW010682). Marieke van der Zalm is part of a career development grant from the EDCTP2 programme supported by the EU (99726 TB-Lung FACT TMA 2015 CDF-1012) and the Fogarty International Center of the National Institutes of Health under Award Number (K43TW011028).

## Declaration of Competing Interest

The authors declared no conflict of interest.
